# Evaluation of Olive Oil Quality with Electrochemical Sensors and Biosensors: A Review

**DOI:** 10.3390/ijms222312708

**Published:** 2021-11-24

**Authors:** Alexandra Virginia Bounegru, Constantin Apetrei

**Affiliations:** Department of Chemistry, Physics and Environment, Faculty of Sciences and Environment, “Dunărea de Jos” University of Galaţi, 47 Domnească Street, 800008 Galaţi, Romania; alexandra.meresescu@ugal.ro

**Keywords:** sensor, biosensor, e-tongue, olive oil, voltammetry, phenolic compounds

## Abstract

Electrochemical sensors, sensor arrays and biosensors, alongside chemometric instruments, have progressed remarkably of late, being used on a wide scale in the qualitative and quantitative evaluation of olive oil. Olive oil is a natural product of significant importance, since it is a rich source of bioactive compounds with nutritional and therapeutic properties, and its quality is important both for consumers and for distributors. This review aims at analysing the progress reported in the literature regarding the use of devices based on electrochemical (bio)sensors to evaluate the bioactive compounds in olive oil. The main advantages and limitations of these approaches on construction technique, analysed compounds, calculus models, as well as results obtained, are discussed in view of estimation of future progress related to achieving a portable, practical and rapid miniature device for analysing the quality of virgin olive oil (VOO) at different stages in the manufacturing process.

## 1. Introduction

The olive tree (*Olea europaea L*.) is native to the Mediterranean basin and parts of Asia, being cultivated, at present, throughout the world for olives and olive oil [1]. Olive oil is considered one of the healthiest food fats, since its consumption has been associated with reducing cardiovascular diseases through decreasing the LDL-cholesterol fraction [2,3], also having an antioxidant [4,5], anti-inflammatory [6], antimicrobial [7,8] and antitumoral [9,10] effect.

Vegetable oils represent an important part of a balanced, healthy diet. The fat fraction of vegetable oils (of up to 90% of the total composition weight) has a much higher energetic value by comparison with that of proteins and carbohydrates [11,12]. Moreover, oils can provide the body with liposoluble vitamins, simultaneously being an important source of energy. The nutritional value of vegetable oils is heavily correlated to their quality. There are several international institutions, such as the Food and Agriculture Organisation of the United Nations and the European Anti-Fraud Office, who develop legislative initiatives for consumer protection against oils which have a questionable quality or are adulterated [11].

Thus, various standards were introduced for the manufacturing process, adequate labelling and quality parameter quantification of vegetable oils such as olive oil. The majority of vegetable oil methods of analysis have certain disadvantages, such as implementation restricted to well-equipped laboratories with specialised personnel and high costs [13,14].

Each method of analysis usually targets a certain quality parameter (value of the peroxide index, value of the anisidine index, fatty acid composition, etc.), yet more tests and various measurements are necessary to correctly evaluate quality [15].

These limitations have led to the increase of interest in research on developing new analytic techniques in oil analysis. For instance, fluorescent spectroscopy was used to detect extra virgin olive oil adulteration with other types of oil (sunflower oil or peanut oil) [16,17,18,19]. The FTIR spectrometric method, together with chemometric techniques, offers another opportunity to evaluate the authenticity of extra virgin olive oil [20] or to estimate the tocopherol, tocotrienol and plastochromanol-8 content in vegetable oils [21]. Furthermore, various strategies of classifying olive oil through FTIR and Raman spectroscopy have been applied [22]. The main disadvantage of authenticating olive oil using spectroscopic techniques such as FTIR and Raman is the reduced number of vegetable species used to construct olive oil and other edible vegetable oil blends. Many researchers use a small set of oils to produce blends, sometimes even using only one type of olive oil or a limited number of edible vegetable oils (not olive oil) in the various blends produced [22,23].

Gas chromatography, combined with mass spectrometry, has been used to determine the ratio between stable isotopes, a valuable parameter in identifying vegetable oil [24]. Moreover, high performance liquid chromatography with atmospheric pressure chemical ionisation mass spectrometry was applied to classify various vegetable oils depending on their triglyceride composition [25]. In another study, high performance liquid chromatography (HPLC) with a diode array detection (DAD) and fluorescence detection (FLD) system was used to identify phenolic fractions which are representative for more types of extra virgin olive oils, in view of authenticating monovarietal olive oils [26].

Sometimes, HPLC separation is not done adequately, and may take a relatively long analysis time due to the complexity of the secoiridoid species which interact with the mobile phase. This problem may be overcome through using more sophisticated instruments such as UHPLC coupled to MS/MS [27] or innovative electrochemical methods which allow the quantification of parameters, such as polyphenol concentration in olive oil.

More researchers focused on this approach, using electrochemical sensors and biosensors to monitor quantities of antioxidant substances and the quality of olive oil, respectively [28,29,30,31,32,33]. The technology for developing sensors and biosensors allows the production, at low costs, of numerous devices which are easy to use and have good reproducibility.

The analysis of olive oil samples using a screen-printed sensor is achieved after a liquid–liquid extraction, which takes much less time (several minutes) than the standard analytical methods (HPLC or spectrophotometry with Folin–Ciocalteu reactive) [28]. In the case of biosensor use, the results are based on the catalytic activity of the immobilised enzyme (tyrosinase) on the surface of the electrode, and the main advantage in using this device is that previous extraction is not necessary since the biosensor is capable of functioning in an organic solvent. The pre-treatment of the sample may thus be eliminated, which reduces the analysis time [28].

More recent research has focused on applying sensor arrays—more specifically electronic nose, electronic tongue or even electronic eye—to differentiate between edible and non-edible olive oils [34,35] or to evaluate the quality of olive oil in relation to rancidity degree and validity period [36].

Along these lines, Dias et al. [37] used a potentiometric electronic tongue to classify the extra virgin olive oils obtained from different olive varieties, while voltammetric electronic tongues were used to quantify the total polyphenol content [38] and the acidity coefficient [39] in extra virgin olive oils. Regarding the electronic eye (e-eye), Cano Marshal et al. [40] applied a computer vision system to estimate impurities in the olive oil samples. In a limited number of published articles, sensor arrays—which represent the electronically detected senses—were simultaneously applied in the detailed characterisation of olive oils [41,42,43].

The present paper aims at highlighting the importance of certifying the authenticity of olive oil through analysing its main quality parameters, but also at highlighting the electrochemical methods used to detect olive oil adulteration. The following sections describe various electrochemical sensors, sensor arrays and biosensors used in evaluating the quality of olive oil. Table 1 presents part of the sensitive devices reported in the specialised literature, the main parameters analysed and the detection limits obtained in the qualitative analysis of olive oils.

## 2. Olive Oil—Determination of Quality Parameters

**Figure 1:**
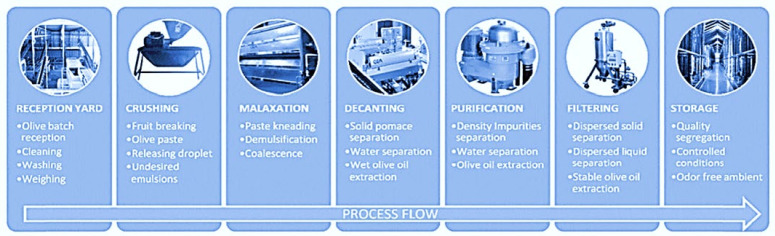
Process scheme for virgin olive oil production. Published from [56] with the permission of the publisher.

Virgin olive oil (VOO) is obtained from fruit (olives), through extraction, using mechanical methods only (crushing olives in a hammer crusher). The olive paste obtained is then mixed, and the olive oil is separated using presses or centrifugal devices. Before storage, the oil obtained is percolated or centrifuged for clarification, resulting in pure virgin olive oil [55]. Figure 1 shows the process scheme for virgin olive oil production.

Olive oil has, in its composition, a fraction with triglyceride content (up to 90–99% of the olive oil) and a non-saponifiable fraction (0.4–5% of the olive oil). The latter includes more phenolic compounds [57,58], the olive oil consumption thus contributing to the daily intake of secoiridoids, absent in other food groups such as fruit, vegetables and cereals [59,60].

The decomposition products of major phenolic constituents—namely oleuropein and ligstroside and aglycon together with hydroxytyrosol and tyrosol—form the majority of the phenolic fractions in olive oil [61,62].

These compounds have a different o-phenolic structure, and are important biomarkers for authenticating the correct preservation of olive oil.

In vitro studies have shown that the phenols in virgin olive oil have antimicrobial, anti-inflammatory or chemo preventive effects on gastric or intestinal cells, depending on the dose [63,64]. Furthermore, there are studies which show that the presence of tyrosol and hydroxytyrosol have an osteoprotective effect, stimulating calcium absorption [65] and osteoblast cell proliferation [66].

Consequently, the beneficent properties related to the intake of phenolic compounds as a result of olive oil consumption are dependent on: the concentration of these compounds [67], the olive variety, the geographic origin and climate conditions [68,69], the agronomic practices [70,71], olive tree ailments [72], the maturity of olive tree fruits harvested [73], olive oil extraction and filtering [69,74], production processes (malaxing, grinding and crushing) [75], storage conditions and time from harvesting [60], etc.

There are also other analytical parameters of great importance for the classification, characterisation and control of virgin olive oil quality, frequently quantified before storage, among them: acidity coefficient [76], bitter taste coefficient (K_225_) [77] and oleic acid–linoleic acid ratio [78].

The acidity coefficient indicates the degree of hydrolytic deterioration in free fatty acids. Oils with an acidity >2% are not recommended for direct consumption, and have to be refined. Visible spectroscopy or near infrared spectroscopy (VIS/NIR) [79], as well as fluorescence spectroscopy [19] are adequate and frequently used methods for determining the acidity coefficient in olive oil.

K_225_ is a chemical index related to the bitter taste of olive oil [80]. A strong bitter taste is not well tolerated by consumers.

**Figure 2:**
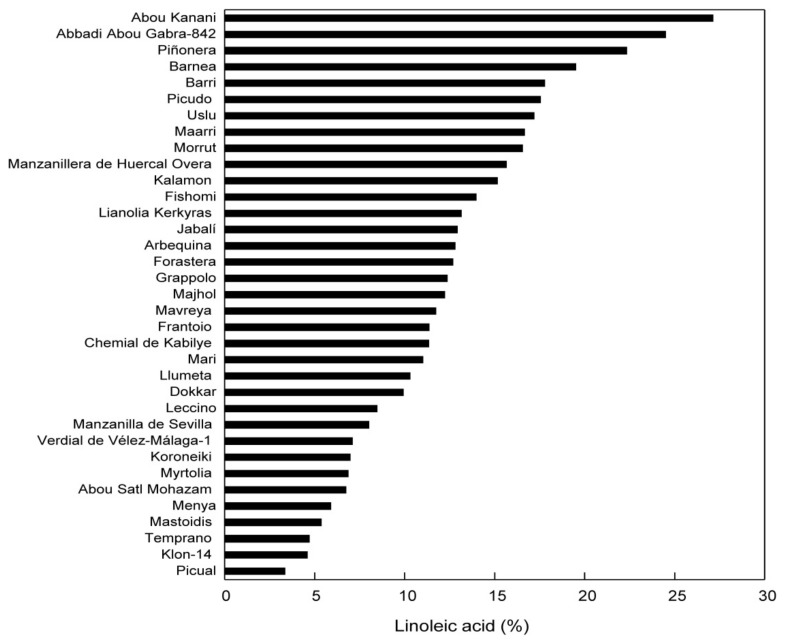
Percentage of linoleic acid in Core-36 oils. The data represent the average of three replicates for the same sample to be analysed. SD is <3% [81] in all cases.

The oleic acid–linoleic acid ratio is estimated based on the profile of the fatty acids, and is calculated as percentage ratio between monounsaturated fatty acids and polyunsaturated fatty acids. This parameter is very important for the market authorisation of the product. High MP values indicate oils with high stability [78]. This parameter was estimated by L. Hernandes et al., in a paper on highlighting the differences between olive varieties in view of improving the quality of olive oil [81]. Figure 2 presents the percentage of linoleic acid for several olive oil varieties.

### 2.1. Phenolic-Biomarker Compounds for Olive Oil

The phenolic compounds in virgin olive oil may be classified into five main categories [82]:

(a) Phenolic acids;
(b) Phenolic alcohols, such as 3,4-dihydroxipheniletanol (3,4-DHPEA or hydroxytyrosol) (3,4-dihydroxyphenyl ethanol) and 4-hydroxyphenil ethanol (p-(hydroxyphenyl) ethanol) (4-HPEA or tyrosol);
(c) Secoiridoids, such as the dialdehyde form of elenolic dicarboxymethyl acid linked to hydroxytyrosol (3,4-DHPEA-EDA) (dicarboxymethyl 21 elenolic acid linked to hydroxytyrosol), called oleacein, and the dialdehyde form of tyrosol elenoliclegate dicarboxymethyl acid (p-HPEA-EDA), called oleocanthal [60], 3,4-(dihydroxyphenyl ethanol) elenolic acid (3,4-DHPEA-EA), called isomer of aglycon oleuropeine, and p-(hydroxyphenyl)ethanol elenolic acid (p-HPEA-EA) or aglycon ligstroside;
(d) Lignans, such as (+)-1-acetoxypinorezinol and (+)-pinorezinol;
(e) Flavones, such as apigenin and luteolin [82].

**Figure 3:**
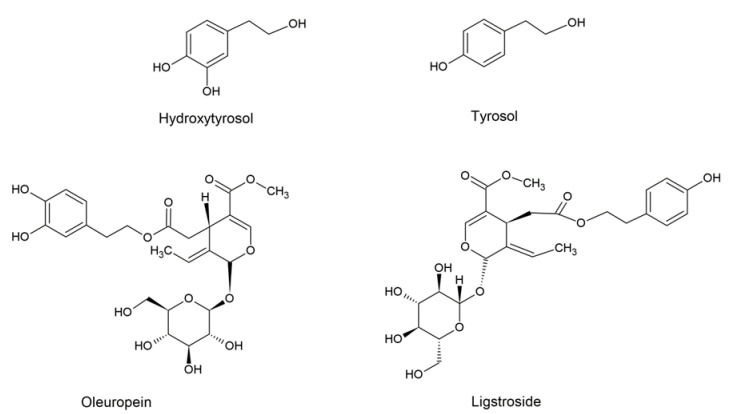
Chemical structures of the main phenolic compounds in olive oil.

The chemical structures of the main phenolic compounds in olive oil are shown in Figure 3.

Following the beta-glycosidase catalysed enzymatic hydrolysis which appears in the olive tree fruit, glycosidase secoiridoid derivatives are obtained, more specifically oleuropein, dimethyloleuropein and ligstroside. The presence of phenolic compounds in an adequate percentage guarantees taste and aroma, but also the stability of the oil during storage [83].

For instance, oleuropein increases during olive tree fruit maturation, being an indicator for phenolic maturation. There is, however, a significant difference between the phenolic maturation and the industrial maturation of oil [84]. 3,4-DHPEA-EDA, derived from oleuropein, is responsible for the bitter taste, while p-HPEA-EDA, derived from ligstroside, is responsible for the pungent taste, both positive characteristics of olive oil [85,86].

The presence of phenolic compounds gives olive oil multiple biological activities, such as an increase in plasmatic antioxidant activity and anti-inflammatory activities [87,88]. For example, oleocanthal has an intramolecular activity, similar to that of ibuprofen [89], and 3,4-DHPEA has proved useful in reducing the effects of TNF-alpha and 1 B interleukin with proinflammatory action [90]. Taking into account these actions which are beneficial for the human body, the European Food Safety Authority (EFSA) declared that virgin olive oil is a beneficial product for the human body if it meets two conditions. Olive oil must contain at least 5 mg of hydroxytyrosol and its derivatives, that is, oleuropein and tyrosol in 20 g of oil, and this statement must be correlated with the specification that the benefits for human health are obtained only if 20 g of oil are consumed each day [91]. Thus, the detection specific to phenolic compounds may be considered a conventional parameter, very widely used in the evaluation of olive oil.

The antioxidant activity of phenolic compounds in olive oil depends on their quantity and variety. It can be evaluated through chemical methods such as DPPH (method for the scavenging of the 2,2-diphenyl-1-picrylhydrazyl radical) [92], ABTS (method for the scavenging of the 2,2′-azino-bis(3-ethylbenzothiazoline-6-sulfonic acid) radical [93] and ORAC (method for the scavenging of the oxygen radical) (oxygen radical absorbance capacity) [94] or through accelerated oxidation methods such as OSI (Oil Stability Index) [95] or the Rancimat test [96].

**Figure 4:**
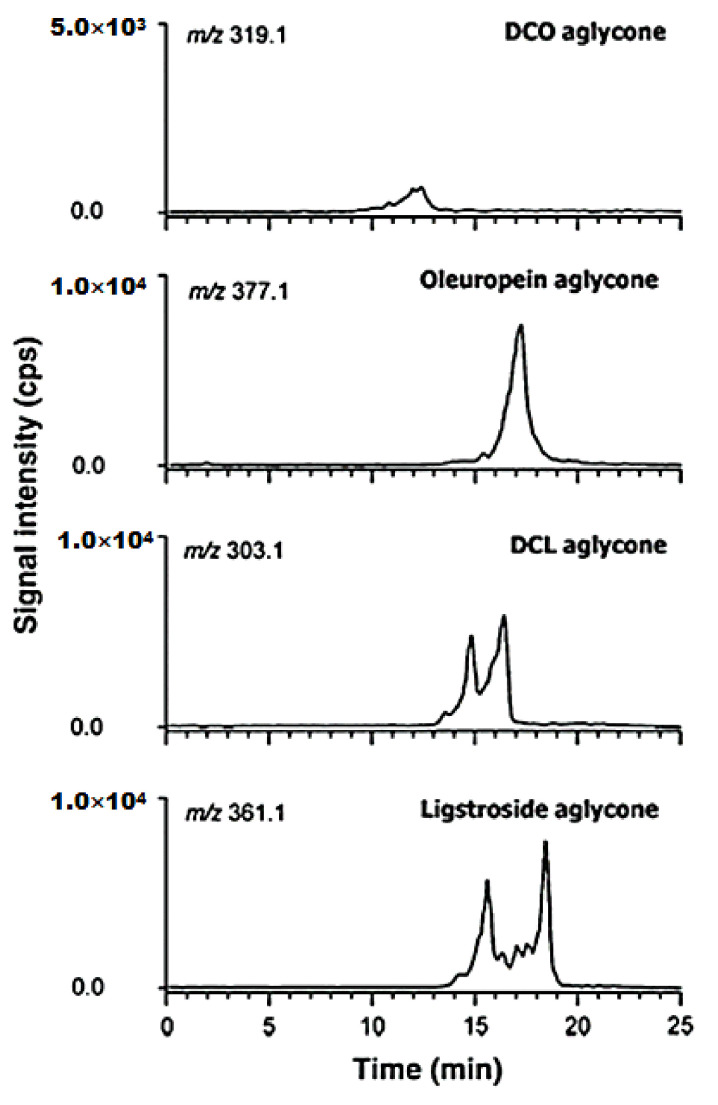
Chromatograms obtained for secoiridoid compounds after the HPLC–ESI(−)–MS analysis of virgin olive oil extracts. Published from [97] with the permission of the publisher.

One of the simplest methods of evaluating the total concentration of phenolic compounds is the colorimetric method based on the Folin–Ciocalteu reactive. However, the method is less specific in comparison with chromatographic methods, which leads to differences between the results obtained [82]. Nevertheless, there are limitations even in the case of high-performance chromatography, the method proposed by the International Olive Council. It was noticed that, when tyrosol is used as external standard, and the quantification is done depending on this compound, oleacein and oleocanthal cannot be estimated correctly due to the differences which occur at the level of the UV response factor and of the molar masses [97]. To avoid these limitations, calibration curves for other commercial standards—such as oleuropein, oleacein or oleocanthal—could be achieved. Such a procedure was applied by R. Pascale et al., who analysed se-coiridoid aglycons using reverse phase liquid chromatography coupled with linear quadrupole ion-trap mass spectrometry with electrospray ionisation in the negative mode (Figure 4). Thus, the total content of decarboxymethyl oleuropein aglycons, de-carboxymethyl ligstroside, ligstroside and oleuropein, expressed depending on the oleuropein standard [98].

### 2.2. Other Bioactive Compounds

In addition to the hydrosoluble phenolic compounds, there are other lipophilic compounds, such as vitamin E, which are relevant from a nutritional point of view. In olive oils, α-tocopherol is mainly responsible for significant concentrations of vitamin E, which can vary from 1.2 and 43 mg/100 g [62,98].

Therefore, tocopherols can also represent quality markers for virgin olive oil [99]. Tocopherols contribute to increasing the stability of the oil alongside phenolic compounds, and play an important role in the protection of the live cellular membrane and in reducing the oxidant reactions on lipoproteins [90]. In virgin olive oil, the analysis refers mainly to α-tocopherol, since the quantities of β- and γ-tocopherols are negligible [100]. Along these lines, more quantification methods were used, including HPLC coupled with mass spectrometry [101], near infrared spectroscopy (NIRS) [102] or reversed phase high pressure liquid chromatography coupled with diode array detection (RP–HPLC–DAD) [103].

The colour of virgin olive oil may vary from yellow-green to gold, depending on the level of olive maturation [104]. Colour is an organoleptic parameter of VOO, which influences the consumer’s perception on the quality of the product. Chlorophylls and carotenoids are the main pigments responsible for the colour of the oil. During VOO storage, chlorophyll undergoes specific degradations which lead to modifications in the pigment [105]. Regarding carotenoids, lutein is the major component, followed by β-carotenoid. Lutein has an antioxidant effect and contributes to preventing macular degenerescence related to age and cataract formation [106], and the presence of chlorophyll is associated with chemo preventive actions against cancerous agents [107].

There are more factors which can influence the composition of chlorophyll and carotenoids, among them: the variety of olive fruits, the geographic origin, the ripeness degree of the fruit, the extraction process and the oil storage conditions [108]. Furthermore, chlorophylls and carotenoids greatly influence the stability of VOO due to that they are antioxidant in the dark and pro-oxidant in the light [109,110]. Recently, a group of researchers reported the determination of chlorophylls and carotenoids in olive oil through the colorimetric method. Specific extinction coefficients for pheophytin (the major component of the chlorophyll fraction) and for lutein (the major component of the carotenoid fraction) were taken into account [110].

## 3. Olive Oil Adulteration

The nutritional and biological benefits of VOO confer it high quality and commercial value. Limited production, high price and increased demand for this healthy, good tasting oil render it susceptible of intentional adulteration with low quality vegetable oils in view of obtaining substantial financial gains [111]. VOO is frequently counterfeited with sun flower oil [112], rapeseed oil [113], peanut oil [114], corn oil [115], walnut oil [116] and soybean oil [117]. Another way to adulterate VOO refers to mixing the oil obtained through cold pressing and simple filtering with a refined oil. Through this procedure, observing a series of stages (such as neutralisation, clarification and smell absorption—usually involved in refining the oil) is avoided. Thus, a lower quality oil is obtained, with trans fatty acids in reduced quantities [118].

The determination of VOO authenticity, regulated by the International Olive Council [119], requires a multitude of analytical methods and techniques. Adulteration of VOO with lower cost and lower quality vegetable oils displeases the consumer and can even cause health problems, especially if it is bought for its nutritional benefits [111].

The EU Commission, the International Olive Council and the Codex Committee for fats and oils regulate and supervise the quality of VOO through imposing certain limit values for the quality parameters of olive oils [120]. For instance, VOO, mixed with soybean oil or walnut oil, will have fatty acids under 5% [118]. These organisations have also described official methods of controlling the quality of VOO. Detecting adulteration of VOO may be done either through quantifying a certain specific chemical marker, such as tocopherol or oleuropein, or through determining the total chemical composition of the sample analysed [111]. Nevertheless, certain recommended methods (such as chromatographic techniques) are laborious, complicated, use expensive and toxic chemical substances, and require stages of sample preparation before analysis [120]. On the other hand, spectrometric, spectroscopic methods and RMN are considered simpler, more rapid and efficient. Recently, electrochemical techniques based on sensors, associated with chemometric instruments or not, represent rapid, portable and reproducible alternatives for the chemical and sensorial analysis of VOO [62,121]. The next section is intended to describe and to analyse the most recent electrochemical techniques, the sensitive devices developed, and the results obtained in connection with detecting VOO adulteration.

## 4. Electrochemical Methods for Evaluating the Quality of Olive Oil

Through the years, electrochemical instruments have been used to analyse various aspects of olive oil authenticity. Firstly, the literature reports sensors and sensor arrays used to evaluate geographic origin, olive oil variety and organoleptic characteristics—important aspects, especially from an economic point of view [122,123,124,125,126,127,128].

Another area of research where electrochemical methods were used was monitoring olive oil quality and resistance to oxidation during storage [129], but also establishing and evaluating the validity period of olive oils [36,43].

The sensor arrays have successfully identified VOO adulteration with other vegetable oils or with low quality olive oils through parameter quantification [30,130,131,132]. The intensity of the sensorial defect predominantly perceived (DPP) is a parameter which analyses the acid, bitter or salty taste of olive oil, and was quantified, in several studies, with the aid of e-tongue [31,133,134,135,136].

Voltammetric [32,44,128], potentiometric [137,138] and amperometric [49,139] methods were also used to determine the total content of polyphenols, flavonoids and phenolic acids in olive oils.

### 4.1. Electrochemical Sensors for the Detection of Phenolic Compounds in Olive Oil

O-diphenols are easily oxidised during inappropriate or long-term storage of olive oil. The quantity of o-diphenols in olive oil has an important role since it endows this food product with an antioxidant potential, beneficial for the health of the human body [140,141]. Developing several sensitive devices for the selective detection of the antioxidant fraction is therefore important for valorising and evaluating VOO.

Phenolic compounds are easy to determine and quantify with the aid of carbon-based sensors [142], and the electrochemical responses offer information on the reaction mechanism and the functional properties of the substance [143,144,145,146].

**Figure 5:**
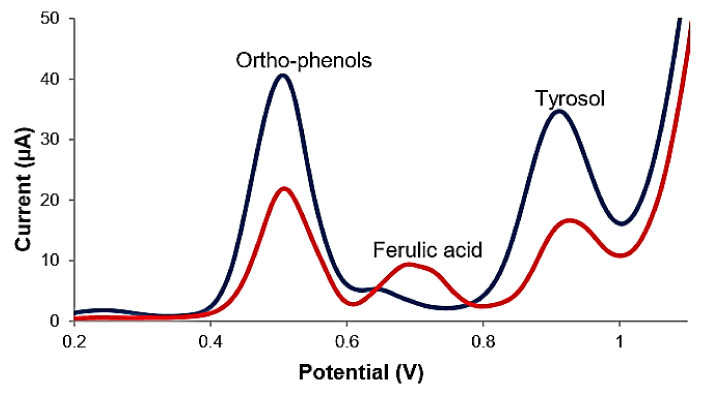
DPV voltammograms of a mixed standard solution formed by hydrophilic phenolic compounds of 10 mg L^−1^ concentration in HCl 0.1 M (red) solution and a virgin olive oil sample in HCl 0.1 M solution after the RP DLLME (blue) procedure. Published from [32] with the permission of the publisher.

In a recent study, phenolic compounds—such as oleuropein, tyrosol, hydroxytyrosol, caffeic acid and ferulic acid—have been analysed from refined olive oil, virgin olive oil and extra virgin olive oil samples, with the aid of a carbon-based screen-printed sensor, applying differential pulse voltammetry. The electrochemical method was combined with reversed phase dispersive liquid–liquid microextraction (RP DLLME) and compared with the Folin–Ciocalteu spectrophotometric method, with close results being obtained. It was noticed that the oxidation of ortho-phenols was achieved at very close potentials, while the mono-phenols underwent more obvious oxidations, at different potentials. The influence of interferents was also studied, varying, in turn, the concentration of one of the compounds detected (caffeic acid and tyrosol). Worth mentioning is the fact that, in the case of tyrosol, the oxidation process was deposited on the surface of the electrode, replacing the sensor with each analysis being necessary. For quantification from real samples, caffeic acid and tyrosol were selected. The lowest content of hydrophile phenolic compounds corresponded to the samples of refined olive oil, and the highest concentration to extra virgin olive oil samples. Figure 5 shows the benefit brought by RP DLLME in detecting hydrophilic phenolic compounds in a virgin olive oil sample.

Therefore, the method proposed involves a simple technique of preparing the sample and using an unmodified screen-printed sensor with commercial availability and reduced cost, being adequate to differentiate the refined olive oil from the extra virgin olive oil. The detection limit obtained had reduced values, which confirms the advantages of the electrochemical method [32].

The modifications made to the electrodes can have multiple benefits regarding the detection of polyphenols, but there is the possibility of certain limitations, as has been presented in the previous study, related to the contamination of the electrode surface due to the polymerisation of the oxidation product and the deposit of an insulating layer which will disturb a future reaction [147,148]. Consequently, the functioning of the electrode is an important stage, and the nanomaterials used must be adequately selected in view of increasing the sensitivity and selectivity of the sensor developed.

Such an approach was selected by M. Del Carlo et al., who used molybdenum (VI) to modify the modified carbon electrodes. Researchers have reported the formation of certain complexes between sodium molybdate and o-diphenols, thus influencing the selectivity of the sensor. The immobilisation of the mediator on the surface of the electrode was also achieved using various types of single walled or multi-walled carbon nanotubes. The best sensor from the point of view of selectivity, sensitivity and stability was the Mo-MW-CNT-NH_2_ modified electrode (multi-walled carbon nanotubes functionalised with amino groups). The amperometric analysis through flux injection was used to determine the content of three o-diphenols (catechol, caffeic acid, hydroxytyrosol) and three mono phenols (tyrosol, hydroxybenzoic acid, ferulic acid) from phenolic extracts obtained from olive oil samples. Following the optimisation of the potential it was noticed that the maximum sensitivity was obtained for hydroxytyrosol. The amperometric method was compared with the spectrophotometric test using the standard catechol solution. A slight discordance was noticed between the results of the two methods, a supplementary dilution of the olive oil samples (from 1/100 to 1/200) being necessary for the linearity range of the calibration curve to correspond. Using the mediator either in solution or in immobilisation, the detection of o-diphenols was achieved, with good linearity in the parts per million range [54].

In conclusion, the intrinsic sensitivity of the electrochemical measurement is capable of decreasing the influence of potential interferents, which leads to a more precise estimation of o-diphenols in real samples. The electrochemical method and the sensor developed proved to be adequate in monitoring o-diphenols in olive oil samples due to test automation sensitivity and selectivity [54].

In another study, T. A. Enache and his team developed an electro-analytical method to determine the total content of ortho-phenol in virgin olive oil (VOO) with increased sensitivity and reproducibility. The ortho-phenol content depends on the VOO freshness in connection with the quantity of hydroxytyrosol (HT equivalents). Screen-printed electrodes were used applying cyclical voltammetry to study the oxidation of catechol, phenol, and hydroxytyrosol (HT), and tyrosol, caffeic acid and ferulic acid. The oxidation of ortho-phenols and mono-phenols takes place following different mechanisms at different potentials. Using screen-printed electrodes and square waved voltammetry a detection limit of 0.40 μM was obtained for HT. The electro-analytical method developed was applied to quantify the ortho-phenol content in VOO with different storage durations. The HT equivalent determined for the two-year-old VOO sample was 3 mg/kg, for one-year-old samples was 6–7 mg/kg, and for a fresh VOO sample it was 30 mg/kg. The influence of the components of a VOO matrix on the response obtained on the oxidation of the HT standard was also studied. The HT standard underwent a recuperation in the 78–93% interval, with RSD 1–3% for two concentration ranges. The results obtained show that the procedure proposed can be applied to evaluate the freshness of VOO [44].

In 2019 a new hybrid nanomaterial was reported; it was made up of carbon black and molybdenum disulphate and was used to modify a screen-printed electrode (CB-MoS_2_/SPCE) in view of detecting ortho-diphenols of the oleuropein and hydroxytyrosol type in extra virgin olive oil and in real samples.

By comparison with individual nano materials, CB-MoS_2_/SPCE shows an improved electronic transfer, increased electric conductivity and improved electro-catalysis. The sensor has an increased sensitivity, without the application of adsorption voltammetry being necessary, as well as reduced time of analysis and increased resistance to contamination. The electrochemical method used was differential pulse voltammetry. As a result of the analyses, OLEU can be detected in the concentration range from 0.3 to 30 μM with an LOD of 0.1 μM, and HYT in the 2–100 μM range with an LOD of 1 μM. The results regarding the quantitative electrochemical determination of o-diphenols in EVOO were close to those obtained with HPLC–UV [47].

The study can open new perspectives for hybrid carbon nanostructures combined with sulphates of transitional metals to develop sensors destined for evaluating the quality of olive oils.

Carbon black was used in the following study also to construct a sensitive device through depositing a nano material dispersion on a polymeric support (aPMMA methyl polymethacrylate), in view of a selective electrochemical detection of certain antioxidant classes such as ortho-diphenols and mono-phenols. After optimising the optimum quantity of CBNP dispersion used for deposit on aPMMA support, the electrodes were characterised through multiple methods—electrochemical impedance spectroscopy, cyclical voltammetry, atomic force microscopy, and Raman spectroscopy—thus confirming the carbon black film imprint on the polymer surface with very good conductivity. CBNP electrodes were used to detect ortho-diphenols and mono-phenols having good reproducibility. The electrochemical methods used were cyclical voltammetry and differential pulse voltammetry. The selective electrochemical indices (EI) for ortho-diphenols and mono-diphenols contributed to evaluating the content of phenolic type antioxidants in olive oils. The parameters were calculated using hydroxytyrosol and tyrosol as standards. The ortho-diphenols and mono-phenols concentrations obtained are in the expected range, demonstrating that the method can be applied for the analysis of real olive oil samples. In real samples a good repeatability was obtained both for ortho-diphenols and for mono-phenols with RSD < 6% and RSD < 15 %, respectively, allowing the simultaneous quantitative analysis of both classes of compounds [48].

Antioxidants can be considered a particular case in which the dose–effect correlation is not necessarily linear. For example, in the case of olive oil, CODEX STAN 33-1981 allows a maximum of 2000 mg/kg of alpha-tocopherol. Additions of α-tocopherols are allowed in view of re-establishing the natural content lost in the refining process, however without surpassing the admissible limit mentioned previously.

The α-tocopherol content in olive oils has become a topic of interest for I. Vasilescu et al., who developed an electrochemical method based on the 2,2-diphenyl-1-picrylhydrazyl (DPPH) free radical to determine this antioxidant compound. Differential pulse voltammetry was used as the measurement technique, while the electrochemical process was registered with a Pt screen-printed electrode. A decrease in the spot current intensity corresponding to the 2,2-diphenyl-1-picrylhydrazyl radical registered at a potential of +160 mV in the presence of α-, β- and δ-tocopherol was noticed, but also when the olive oil samples were analysed.

The results obtained using DPV were close to those obtained through HPLC coupled with fluorescent detection. The electrochemical method reported is rapid, easy to use, efficient and accessible to be used as an alternative to the spectrophotometric method for the evaluating the antioxidant properties of olive oil [45].

### 4.2. Electrochemical Sensors for Evaluating the Acidity Index and the Peroxide Index of Olive Oil

**Figure 6:**
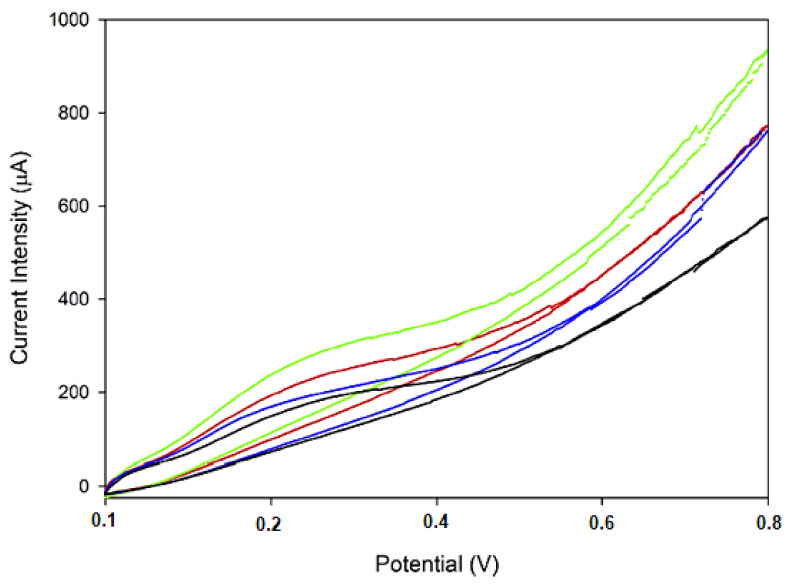
CV of oils obtained from each of the four categories: Leccino (black); Moraiolo (blue); Frantoio (red); Nostrale (green). Published from [46] with the permission of the publisher.

The acidity index and the peroxide index are important parameters in evaluating the authenticity of olive oil. A very recent study was aimed at classifying and differentiating extra virgin olive oil varieties (EVOO) depending on the geographic origin, through measuring these parameters. In this study, the researchers used a screen-printed electrode modified with multi-walled carbon nanotubes and titanium oxide nanoparticles, applying cyclical voltammetry. The modification of the electrodes was carried out through the drop and dry technique, using a suspension made up of multi-walled carbon nanotubes, titanium oxide nanoparticles and a biological ionic liquid (based on choline). The liquid obtained was subjected to sonication before depositing on electrodes. The sensors obtained had a large active surface area, high stability (up to 30 days) and excellent reproducibility. These modified electrodes were used to measure acidity (percentage of free fatty acids), resulting in similar values for the samples analysed with the exception of an oil of the Moraiolo variety. As for the peroxide index, it can vary even in the case of oils similar as variety, therefore it cannot be used as defining parameter to differentiate these products. As a result, the researchers used an enzymatic method (based on lipase), which allows the oxidation of the compounds present in EVOO. The EVOO varieties were classified based on the values of the anodic spot potential registered, the latter being correlated with the concentration of antioxidants in the sample. The potential ranges are represented for every EVOO variety, which leads to the minimising the risk of classifying an unknown sample in the wrong category (Figure 6).

The precise results obtained show that the electrochemical method is valid and feasible, and the fact that electrode modification and sample analysis do not imply the use of organic solvents attests that the technique is ecological and may be used for testing EVOO directly, on site [46].

### 4.3. Electrochemical Sensors for the Sensory Evaluation of the Bitterness and Purgency of Olive Oil

In addition to the benefits for the health of the human body (antioxidant [149], antineoplastic [150], antimicrobial [151]), oleuropein is also responsible for the bitter taste of the olive oil, being an important parameter for evaluating the quality of this food product. The oleuropein content may vary depending on climactic factors, agricultural practices, and extraction techniques, but also on the olive maturation stage [90,152,153]. Consequently, the quantification of oleuropein may offer information regarding the optimum moment for fruit harvesting in view of obtaining superior quality VOO. A lot of research has reported the sensorial evaluation of the bitter taste, but the standard analytical techniques NIR [154], UV-VIS [155], and Raman spectroscopy [156]) are expensive, require a long time and qualified personnel, being avoided as routine analysis of VOO.

The study carried out by K. Morozova et al. proposes a direct, rapid electrochemical method with a high performance in evaluating the bitter taste of EVOO. This parameter was determined through amperometry, using a flux injection system, and the device which registered the signals was a vitreous carbon electrode. The optimum potentials were selected depending on the oleuropein hydrodynamic voltammograms. A total of 32 EVOO samples were analysed from the point of view of the total content of phenol and oleuropein. The results obtained were satisfactory, being correlated with the organoleptic exam performed by experts and with the profile of the phenolic content determined through HPLC [49].

### 4.4. Sensor Arrays. E-Tongue for the Detection of Phenolic Compounds in Olive Oil

In another study of great relevance, an electronic tongue was also used for determining extra virgin olive oil adulteration. To this end, square wave voltammetry, sensors modified with carbon paste, and chemometric methods for interpreting electrochemical signals were used. With the aid of the sensor array, the detection of several phenolic fractions—partly characteristic to other vegetable oil varieties, such as tocopherols and triglycerides–was achieved. However, there are some differences of voltammetric signals in vegetable oils which can be due to individual physical–chemical properties such as viscose. Finally, excellent correlations were obtained through the regression of partial least squares (PLS) between the voltammetric signals and the polyphenol content obtained. PLS-DA and PLS demonstrated the feasibility of detecting adulteration olive oils with other vegetable oils (added in proportions under 10%).

The results of the study indicate the fact that the electronic tongue can be a useful instrument in detecting adulteration olive oils with other vegetable oils [132].

### 4.5. Sensor Arrays: E-Tongue for Evaluating the Acidity Index and the Peroxide Index of Olive Oil

According to international regulations, the acidity index and the peroxide index are determined, in the laboratory, most frequently, by manual titration. However, this type of method cannot be easily used in the oil production process.

As in the case of the previous study, V. Semenov used a multisensory potentiometric system (e-tongue) based on electrodes with membrane, in solid state, to evaluate the various quality parameters of vegetable oils, olive oil included. After the optimisation of the sample preparation procedures, it was shown that the multisensory system developed is capable of distinguishing between vegetable oil varieties.

The quality indicators potentiometrically analysed were the peroxide index (PI), the p-anisidine value (p-AV) and the total concentration of tocopherols (TT). Despite using a limited number of samples, the multisensory system can recognise the types of samples and a potential adulteration through determining several very important quality parameters [11].

A new portable instrument for measuring the acidity of olive oil has been developed using Electric Impedance Spectroscopy (EIS), which is suitable for making a portable, simple, low-cost instrument that can bring benefits to olive oil producers. The technique was validated with a set of 55 olive oil samples. For measurements, two different oil emulsions were used and compared: one based on hydroalcoholic solution (60% ethanol, 40% distilled water) and another one based on distilled water. The portable instrument was based on a system of built-in stainless-steel sensors, and the detection technique EIS allowed in situ rapid acidity measurements. The results showed that the electrical conductivity of the emulsion based on hydroalcoholic solution is an important parameter for estimating the acidity of olive oil, having an optimal accuracy (R^2^ = 0.9308). The experiments carried out in distilled water-based emulsions, on the other hand, do not show any significant correlation between the acidity of the oil and the conductivity of the emulsion. Instead, these experiments provide information about the peroxide index, the polyphenol content and the filtration technique. Therefore, the presented technique can be implemented in the form of an integrated low-cost electronic system, which can be used to characterise the product on site, in order to reduce the costs of samples transporting [157].

In addition to other quality parameters (the total content of phenolic compounds, tocols levels, oxidative stability), the peroxide index is an important parameter indicating the quality of olive oil. The peroxide index (PI) is a parameter frequently used to evaluate the primary oxidation products (more precisely the amount of hydroperoxides) in olive oil. PI can be used to assess the decrease in oil quality over time, but it must be corroborated with other parameters, because hydroperoxides decompose naturally during storage.

An e-tongue together with a multiple linear regression model (MLRM) coupled with a meta-heuristic simulated annealing algorithm (SA) was used for PI evaluation. The e-tongue detecting system consists of two potentiometric sensor arrays, each containing 20 lipid polymeric cross-sensitive sensors. The results showed that MLRM-SA-e-tongue is able to quantify PI with an analytical accuracy similar to that obtained by the official titration technique [158].

### 4.6. Sensor Arrays. Electronic Tongues for the Sensory Evaluation of the Bitterness and Purgency of Olive Oil

A large variety of chemical sensors is used at present in designing electronic tongues [38]. The sensor array is chosen depending on the chemical nature of the food samples analysed. Regardless of the type of sensors used, an electronic tongue is generally made up of three elements: an automatic harvester (although this is not a mandatory component), an array of chemical sensors with different selectivity, and a software with an algorithm which is appropriate for processing the signal and obtaining adequate results, discrimination and classification [43,121].

The electronic tongue is based on a sensor array with moderate selectivity and cross sensitivity. Thus, each sensor in the array generally offers information on the concentrations of a limited number of analytes [159].

The number of analytes in the array may vary, but usually there are approximately 10–20 sensors. Another advantage of the electronic tongue is the fact that it can function without a reference electrode, since the difference in potential is measured for all the pairs of electrodes in the array. These devices can characterise the samples, not only in as far as the concentration of various analytes is concerned, but also for recognising the nature of the sample analysed [160], which is an important aspect in classifying and differentiating between VOO varieties.

Such a sensor array based on polypyrrole was developed for the analysis of EVOO. The characteristics noticed in the cyclical voltammograms reflect the redox properties of the electroactive compounds (mainly antioxidants) present in the samples pre-treated with extra virgin olive oil. Each sensor in the array has a characteristic electrochemical signal, offering a high degree of cross selectivity. The sensors were constructed through electro-polymerisation, using several doping agents such as potassium hexacyanoferrate (II) (FCN), potassium nitroprusside (NP), phosphotungstic acid (PWA), sulphuric acid (H_2_SO_4_), sodium molybdate (MO) and 9,10-antraquinone, and the sodium salt of 2-sulfonic acid (AQS). The Principal Component Analysis (PCA) and the discriminating analysis solved through the method of partial least squares (PLS-DA) allowed the classification of the six extra virgin olive oils tested depending on their bitter taste [29].

**Figure 7:**
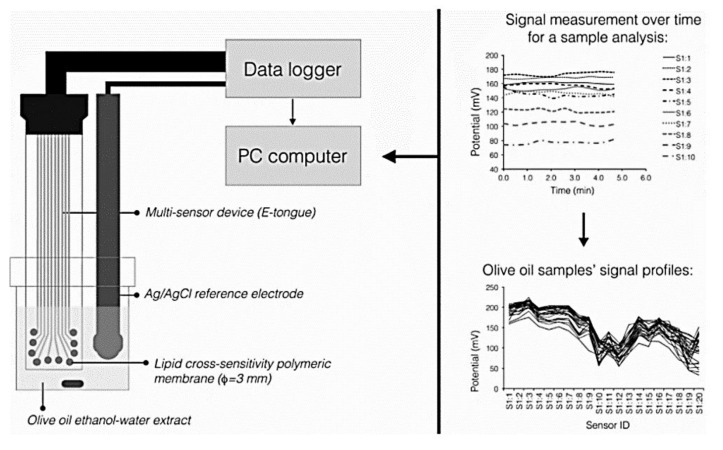
EVOO analysis scheme: electronic tongue (left); potentiometric signals registered for a sample analysed and the profile of the signals registered for the samples analysed (right). Published from [127] with the permission of the publisher.

A.C.A Veloso et al. used as classification criteria not only the degree of bitter taste, but also the fruity aroma and the pungent taste of olive oil. To this end, they used a lipidic polymeric membrane system with cross unspecified sensitivity. To construct the multisensory platform, two potentiometric arrays were used; they had 20 screen-printed sensors, obtained through combining several lipidic and plastifying additives. Initially, the sensor platform was tested using quinine monochlorohydrate standard solutions, optimum sensitivities being obtained depending on the material used. Each sensor was identified with a letter S (for sensor), followed by a code for the sensor array (1: or 2:) and the membrane number (1 to 20, corresponding to various combinations of plasticiser and additive used) (Figure 7) [127].

It is worth noticing that this study used linear models of discrimination for sensor sub-sets, which allowed the correct classification of 91% of olive oils, depending on the bitter taste (leave-one-out cross-validation procedure). The same parameter was evaluated through a K-fold cross-validation procedure, through which it was demonstrated that the electronic tongue correctly classified 80% of the olive oils analysed. Thus, the multisensor device, together with the chemometric methods applied, indicated a correct capacity of testing the intensity of the bitter taste [127].

In 2017, Souihli Slim constructed a potentiometric array with 20 lipidic polymeric membrane sensors, which offered electrochemical responses characteristic to the presence of polar compounds in olive oil samples from Tunisia. This e-tongue could create a mark specific to the olive oil in the area, with large quantities of polar compounds being noticed in the samples analysed. Moreover, the device managed to differentiate olive oils depending on the olive variety, but also on the quality (extra virgin, virgin or lampante olive oils). The linear discriminant analysis coupled with an algorithm for selecting the variables, showed that e-tongue can correctly classify, based on quality, 84 ± 9% of the olive oils and, based on variety, 94 ± 6% (K-fold cross-validation) of the samples [124].

### 4.7. Biosensors for the Detection of Phenolic Compounds in Olive Oil

Biosensors play an important role in food analysis, since they endow the detection method with simplicity, automation, portability and specificity; they also reduce the volume of samples and reactives, and the time and cost of the analysis [161]. The authenticity of food is a challenge frequently addressed by biosensor technology, due to the performance and advantages offered, which have evolved in recent years [162]. The detection of phenolic compounds is of major interest in appreciating food quality, and the use of tyrosinase is useful for detecting phenolic compounds.

Amperometric biosensors based on tyrosinase represent a very simple, convenient instrument for the analysis of phenolic compounds in food products [163,164,165] such as olive oil [166,167].

Tyrosinase (Tyr) or polyphenol oxidase catalyses the oxidation of mono-phenols by molecular oxygen to form o-diphenols, which are then oxidated for o-quinones. These quinones are reduced electrochemically to a low potential, and the current measured is directly proportional to the concentration of the phenolic compound in the sample [148]. There are more strategies by means of which the electrodes are modified with Tyr: physical adsorption, chemical link formation or cross-linking [168]. Furthermore, carbon nanomaterials also play an important role in electroanalysis. The quinones reduced on the carbon particles generate o-diphenols, which are re-oxidated by the enzymes situated in the proximity of the carbon particles, thus allowing an amplified process, considered as an advantage of these immobilising methods [169]. Consequently, a low limit of phenol detection may be detected. In the case of carbon paste electrodes, a large quantity of lyophilised enzyme is used, which may represent a disadvantage of this immobilisation method, since it generates increased costs [170].

This disadvantage was avoided by S. Nadifiyine’s team of researchers, who developed a black carbon paste biosensor based on the immobilisation of a minimum quantity of tyrosinase enzyme (CBPE-Tyr) on the surface of the electrode, and the comparison of the analytical performances of the enzymatic biosensors with those obtained through the standard Folin–Ciocâlteu spectrophotometric method. The electrochemical methods used were amperometry and linear voltammetry. The immobilisation method and the quantity of enzyme used were optimised. The efficient, less expensive method was that of cross-linking using lactaldehyde, sensors with better sensitivity being thus obtained. To determine the phenols in the olive oil samples, caffeic acid was used as standard. It was noticed that CBPE-Tyr has the greatest sensitivity to hydroxytyrosol and tyrosol—the main phenolic compounds in olive oil. Following the experiments, the authors showed that using partially purified tyrosol instead of pure tyrosol does not compromise the quality of real sample phenol determination [50].

Although the main goal of the study was the simple, convenient achievement of a biosensor to determine phenolic compounds in real samples, this device could be used successfully in the routine evaluation of the antioxidant capacity and the quality of olive oil.

A paper published in 2016 presents another technique for immobilising tyrosinase, offering precise results for the biosensor constructed—used for quantifying polyphenols in olive oil. The authors used an auto-assembled ω-mercaptopropyl naphthoquinone monolayer as mediator for incorporating tyrosinase on a gold electrode, obtaining the Tyr/CS/NQ-SAM/GE biosensor. Tyr was added in a mixture with chitosan, a biopolymer which improves considerably the film formation and the biocompatibility with the surface of the electrode. Experimentally, it was noticed that the simultaneous reduction of naphthoquinone in the monolayer mediator generates a stronger current. The calibration curve was achieved in the 0.02–135 μM range for phenol, thus obtaining a sensitivity of 0.0203 μA × μM^−1^. The biosensor was used to determine phenols in olive oil. The optimum selectivity and stability of the sensitive device indicates that this method can be applied to determine the phenol content in virgin olive oils [51].

A complex technology for developing an enzymatic biosensor was reported by F. J. Pavinatto’s research team [171]. The gold (Au) interdigitated electrodes (IDE) with dimensions under 100 mM were screen-printed directly, with an ink jet based on nanoparticles on plastic substrata. The tyrosinase (Tyr) enzyme was used in the active layer of the biosensors, being deposited on the surface of the electrodes innovatively, through rotogravure serigraphy. The composition of the ink was optimised to maintain the optimum activity of the enzyme.

After adding the gravure (with ink containing Tyr), the biosensor was covered with a cellulose acetate film to avoid dissolving. The intensity of the electrochemical signal (obtained through electrochemical impedance) increases linearly with the concentration of a model antioxidant, pyrogallol, allowing the construction of a calibration curve. Taking into account the fact that the sensitivity and the detection limit obtained (5.68 Ω/μM and 200 μM) are much lower than the antioxidant concentration in olive oils [171,172], this biosensor has a high potential for analysing this food product.

**Figure 8:**
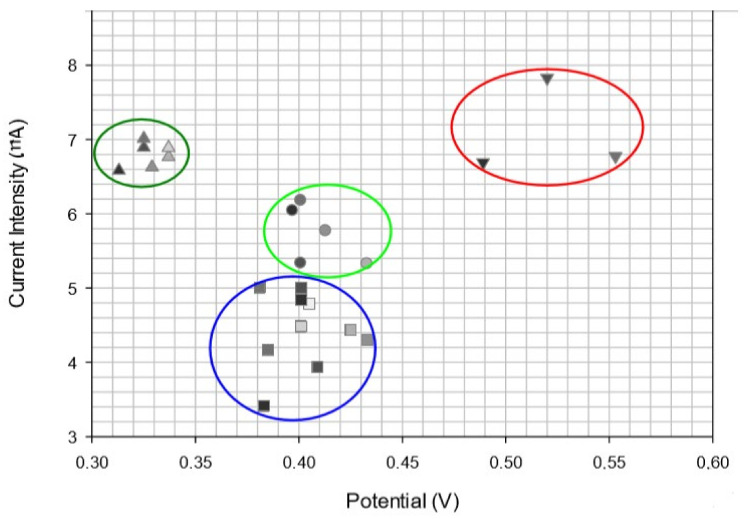
Analysis of extra virgin olive oils from various regions in Italy. Working electrode: GC/MWCNT/[Ch][Phe]/lipase. The points in the chart correspond to the various oil samples. Each geographic region is represented by: ▲ the south of Italy ▼ Tuscany ● Abruzzo ■ Lazio. Published from [175] with the permission of the publisher.

It was demonstrated that the oxidation of antioxidant compounds in the oils was influenced by the hydrolysis reaction of triglycerides by lipase [173]. Therefore, all the oxidative reactions which take place on the surface of the work electrode are due to the antioxidant compounds formed as a result of the action of lipase on the oil. These chemical reactions were exploited with the aid of a biosensor developed through immobilising lipase with an ionic liquid based on choline and phenylalanine on the surface of the screen-printed electrode based on multi-walled carbon nanotubes. Analysing a large number of samples allowed a classification depending on the geographic region where the olive oils are produced. The high values of the potential range and the intensities of the spots obtained could be attributed to the antioxidant composition specific to each oil examined, depending on fruit variety, geographic region and climate [174]. Figure 8 shows that olive oils from the same variety or from neighbouring regions are in the same area of the graph.

Therefore, GC/MWCNT/[Ch][Phe]/lipase can be an opportunity for developing a portable microchip, easy to use in the analysis of olive oil, but of other varieties of samples also.

The electroactive compounds in olive oil can also be determined with the aid of electrochemical biosensors based on DNA. The principal condition is that the analytes have affinity for nucleic acids. The compounds analysed can be detected either directly [173,176], if they are electroactive, or through modifying the electrochemical signal of DNA [174,177]. An interesting study describes the simple, sensitive and rapid determination of oleuropein using a DNA microsensor. The device is based on evaluating the voltammetric behaviour of oleuropein, highlighted on the surface of a carbon paste electrode modified through DNA modification with the aid of chitosan. The electrochemical method by means of which the electrochemical signal of oleuropein was registered was differential pulse voltammetry. After optimising the experimental conditions, a linear concentration range of 0.30–12 μmol × L^−1^ was obtained, with a detection limit of 0.090 μmol × L^−1^, to determine oleuropein. The biosensor proposed was successfully applied to determining oleuropein in an olive leaf extract [178].

Both the technique and the device constructed in this study are considered to be adequate for quantifying this analyte in olive oil, since the mere preparation of samples would require a different pre-treatment.

### 4.8. Biosensors for the Sensory Evaluation of the Bitterness and Pungency of Olive Oil

As has been presented in the previous section, secoiridoid derivatives of hydroxytyrosol and tyrosol are correlated to the bitter taste of olive oil. A study shows that the oils with a slight bitter taste correspond to concentrations of up to 500 mM/kg of secoiridoid derivatives. Moreover, the correlation between the bitter taste and the aldehydic form of aglycon oleuropein (3,4-DHPEA-EA) [179], as well as the correlation between the pungent taste and the quantity of deacetoxi ligstroside aglycon (p-HPEA-EDA) [180] were demonstrated.

The main goal of the following analysis was to research the applicability of enzymatic biosensors to the rapid evaluation of VOO sensorial properties (bitter taste and pungent taste). The performances of the biosensors based on tyrosinase and peroxidase to determine polar phenolic compounds were compared. The enzymes were immobilised on the surface of several carbon-based screen-printed electrodes. The electrochemical results were compared with the data obtained through the HPLC method, considered the reference method. The correlations between biosensors based on tyrosinase and peroxidase, and the phenolic content were high (r 0.82 and 0.87, respectively), which suggests that these biosensors may represent a promising instrument in analysing the total content of phenols in virgin olive oils. The correlation with the sensorial attributes of virgin olive oil was lower, highlighting the complexity of the sensorial perception. The two biosensors indicate selectivity towards various classes of phenolic compounds, influencing the predictability for the bitter and pungent tastes. However, the biosensor based on peroxidase showed a significant correlation with the pungent taste (probably associated with the p-HPEA-EDA content), which is a sensorial parameter responsible for the quality of virgin olive oil [181].

### 4.9. Biosensors for Determining the Degree of Olive Oil Rancidity

As mentioned in previous sections, the main causes of low-quality olive oil include improper handling of olives, oxidation, and improper and prolonged storage. In many cases, the olives are harvested in one country and the oil is processed and bottled in different countries, which implies a long time until the final product is obtained. Under these conditions, olive oil can become rancid or degraded, even before being exported or marketed. Both saturated and unsaturated aldehydes are products of the olive oil oxidation and are highly objective indicators of rancidity of the different olive oils. Therefore, determining the aldehyde content of olive oil could be a parameter for evaluating the product quality. A team of researchers has proposed a new portable, robust, low-cost, enzymatic biosensor capable of determining aldehyde in the finished product, even in a commercial setting. In the optimal conditions, the aldehyde dehydrogenase biosensitive device was able to detect the aldehydes at micromolar concentrations in the presence of NADH as cofactor. Immobilisation of the enzyme was performed on a carbon paste electrode containing Meldola’s Blue, which has a selective oxidative affinity for NADH. Although the design of the biosensor and its responses were satisfactory, there were also limitations regarding the detection of very low concentrations of aldehydes and the need for a protective coating applied before use. However, the enzymatic electrochemical biosensor is an innovative technology that could be improved and used in the olive oil industry [182].

### 4.10. Biosensors for Detecting Contaminants in Olive Oil

It is widely known that evaluating the quality of olive oil does not only presuppose verifying certain concentrations of antioxidants or the possible mixing with an inferior quality vegetable oil, but detecting a possible contamination also. The European Commission introduced olive oil in the category of foods with an associated maximum level of pesticides [183].

A strategy of evaluating the contamination of olive oil with organophosphorus pesticides is proposed by F. Arduni in a relatively recent paper. The sensitive device was constructed through immobilising butyrylcholinesterase on the surface of a black carbon-based screen-printed electrode (BChE/CB-SPE), and the parameters of the amperometric method were optimised to detect paraoxon in the real oil samples after an extraction stage, according to the QuEChERS (Quick, Easy, Cheap, Rugged and Safe) method described in a previous study [184]. The BChE/CB-SPE biosensor showed optimum reproducibility, as well as good analytical performances with a low detection limit (6 ppb) in olive oil extract. The results obtained suggest that the biosensor proposed may be considered as an adequate analytical instrument for analysing contaminants in olive oil [185].

**Figure 9:**
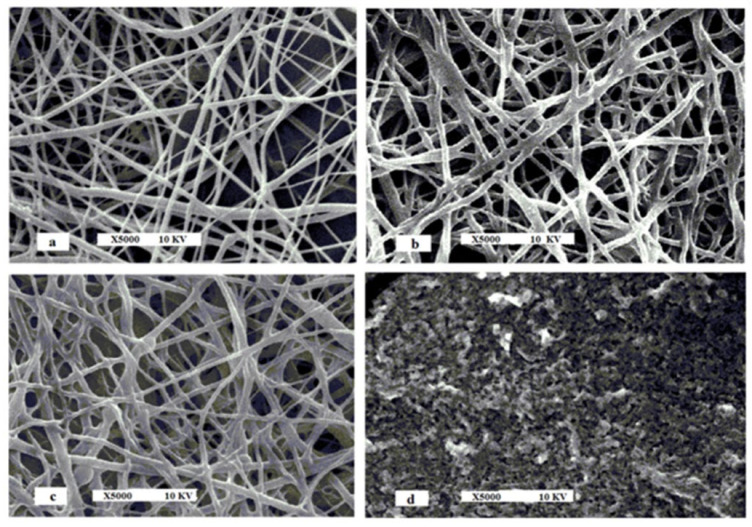
SEM Micrographs of (**a**) electrospun CS-PVA NFM, (**b**) activated electrospun CS-PVA NFM, (**c**) AChE/electrospun CS -PVA NFM, (**d**) AChE/CS-PVA CM (casted electrode). Published from [186] with the permission of the publisher.

Another study presents an ultra-sensitive electrochemical biosensor developed for the rapid detection of another organophosphorus pesticide—pirimiphos methyl—in olive oil. The immobilisation of acetylcholinesterase (AChE) was achieved after the formation of a nanofibrous membrane (NFM) together with chitosan (CS) and polyvinyl alcohol (PVA) through an electrophilic technique. The SEM morphology for each stage of biosensor preparation can be observed in Figure 9.

The biosensor developed showed good performance in detecting pirimiphos methyl, with a detection limit of 0.2 nM, a much lower concentration than the maximum limit of residues admitted—established by international regulations (164 nM). The biosensor was used to detect pirimiphos methyl in olive oil samples after a simple liquid–liquid extraction with recuperation values of almost 100% [186].

## 5. Conclusions and Future Perspectives

Olive oil is a rich source of bioactive compounds with nutritional and therapeutic properties. The aroma and aspect are important organoleptic properties both for the consumer and for producers and traders. These unique characteristics of VOO are correlated with a certain phenolic compound content. For example, oleuropein is responsible for the bitter taste, and p-HPEA-EDA brings a pungent taste. In general, the secoiridoid derivatives and the triglyceride content are parameters for evaluating the quality of olive oils. Adulteration of this food product which is beneficial for human health has become a relatively frequent practice, aimed at obtaining a higher financial profit.

The quantification of various constituent compounds can identify VOO mixes with other, lower quality vegetable oils, but can also facilitate the classification of oil depending on variety, geographic region and climate; it may even suggest the optimum moment for harvest. The standard analysis methods such as chromatography, spectroscopy, and spectrophotometry offer valuable information about VOO composition, but they may have limitations or disadvantages such as lengthy analysis time, numerous reactives, laborious preparation of samples. For instance, the UV-Vis method is predisposed to colour interferences or sample turbidity, and HPLC is an expensive method which requires specialised personnel. The electrochemical methods based on sensors or sensor arrays represent promising alternatives for VOO analysis, being precise, ecological, rapid, and less expensive. The devices described in this review were achieved through innovative techniques, with the goal of identifying and sensitively determining various antioxidant compounds or contaminators in VOO. The carbon nanomaterials are preferred for the construction of sensors, but obvious advantages were noticed when they were associated with mediators which improved electron transfer and sensitivity. As in the case of electrochemical sensors, challenges may occur, especially in relation to contaminating the active surface particularly in the analysis of phenolic compounds, due to the deposit of the antioxidant product, which prevents further oxidations. This impediment can be avoided through adequately functionalising the material.

The sonication stage also has a beneficial influence, since it allows the creation of various stronger links between the nanomaterials and the mediators, offering the sensors better stability and reproducibility. In the case of biosensors, selectivity is a remarkable advantage for detection. In VOO analysis, the devices use, as sensitive recognition elements, tyrosinase, lipase or acetylcholinesterase, but there appears the risk of losing a quantity of enzyme or of substantially reducing its activity when the biosensor is immersed in electrolyte or sample and subjected to electric current. Ways of avoiding enzyme deterioration include either capturing the enzyme in a biocompatible polymeric matrix, or covering the entire active surface with a polysaccharidic membrane (cellulose acetate, for instance). The use of lipidic polymeric membranes can facilitate reactions with substances that influence taste through electrostatic or hydrophobic interactions. The sensor arrays, as well as e-tongue or e-nose, are improved variants which offer multiple analysis criteria and an ample, much clearer characterisation of VOO composition.

Nevertheless, sometimes, the calculus methods associated with the detection method are not very precise, requiring validation through other methods of analysis or the introduction of a large number of samples to create a solid algorithm.

Future research could concentrate on constructing miniature and portable arrays which combine, within the same device, sensors for the analysis of gas, liquid and colour even with enzymatic biosensors capable of selectively, precisely and rapidly determining the constituent compounds of VOO. A lab-on-a-chip type device could be used in the routine analysis of olive oil or in various stages of production—from harvesting to marketing.

## Figures and Tables

**Table 1:** Main electrochemical (bio)sensors, parameters analysed, electrochemical technique applied, and detection limit obtained in olive oil analysis.

(Bio)Sensor	Analyte	Detection Technique	LOD	Ref.
SPCE	caffeic acid	DPV	0.022 mgL^−1^	[32]
SPE	hydroxytyrosol	SWV	0.4 μM	[44]
PtSPE	α-tocopherol	DPV	0.365 μM	[45]
MWCNT/TiO_2_/RTIL/SPE	free fatty acids	CV	-	[46]
CB-MoS_2_	oleuropein hydroxytyrosol	DPV	0.11 μM 1.0 μM	[47]
CBNP	hydroxytyrosoltyrosol	DPV	0.006 μM 0.020 μM	[48]
GCE	oleuropein	Amp	1.58 μM	[49]
CBPE-Tyr	catechol	Amp	6 nM	[50]
Tyr/CS/NQ-SAM/GE	phenol	ChronoAmp	0.019 μM	[51]
SPE	oleuropein	DPV	0.25 ppM	[28]
Tyrosinase-based biosensor	phenol	FIA	4.0 ppM	[28]
PB-GC	peroxide	CV	0.001 mequiv	[52]
GCA	nordihydroguaiaretic acid	CV DPV	-	[53]
Mo-MW-CNT-NH_2_/SPE	catechol	FIA	-	[54]

SPCE: carbon-based screen-printed electrode; SPE: screen-printed electrode; PtSPE: platinum-based screen-printed electrode; MWCNT/TiO_2_/RTIL/SPE: screen-printed electrode based on multilayer carbon nanotubes modified with titanium nanoparticles and choline-based biological liquid; CB-MoS_2_: screen-printed electrode based on carbon black and molybdenum disulfide; CBNP: electrode based on carbon black; GCE: glassy carbon electrode; CBPE: Tyr- biosensor based on carbon black paste modified by immobilisation of tyrosinase; Tyr/CS/NQ-SAM/GE: modified gold electrode with self-assembled monolayer of ω-mercaptopropyl naphthoquinone and tyrosinase; PB-GC: glassy carbon electrode modified with Prussian Blue; Mo-MW-CNT-NH_2_/SPE: screen-printed electrode based on multilayer carbon nanotubes modified with Na_2_MoO_4._; FIA: Flow injection analysis.
